# The Prophylactic Effects of Zintoma and Ibuprofen on Post-endodontic Pain of Molars with Irreversible Pulpitis: A Randomized Clinical Trial

**Published:** 2013-08-01

**Authors:** Mohsen Ramazani, Mahmoud Reza Hamidi, Ali Akbar Moghaddamnia, Nahid Ramazani, Nafiseh Zarenejad

**Affiliations:** a Department of Endodontics, Dental School, Mazandaran University of Medical Sciences, Sari, Iran; b Department of Endodontics, Dental School, Babol University of Medical Sciences, Babol, Iran; c Department of Pharmacology and Toxicology, Medical School, Babol University of Medical Sciences, Babol, Iran; d Department of Pedodontics, Children And Adolescent Health Research Center, Dental School, Zahedan University of Medical Sciences, Zahedan, Iran; e Department of Restorative Dentistry, Dental School, Babol University of e Medical Sciences, Babol, Iran

**Keywords:** Analgesics, Anti-Inflammatory Agents, Ibuprofen, Non-Steroidal, Pain Measurement, Pulpitis, Visual Analogue Pain Scale, Zintoma

## Abstract

**Introduction:**

Post endodontic pain is often linked to the inflammatory process as well as additional central mechanisms. The purpose of the present double-blind randomized clinical trial study was to compare the prophylactic effects of a derivative of Zingiber Officinale, Zintoma, and Ibuprofen on post endodontic pain of molars with irreversible pulpitis.

**Materials and Methods:**

The post endodontic pain of 72 enrolled patients suffering from irreversible pulpitis was assessed after prophylactic use of 400 mg Ibuprofen, 2 gr Zintoma and placebo. Using the Heft-Parker Visual Analogue Scale, the patients recorded their perceived pain before taking the medicament (baseline), immediately after and also at 4, 8, 12, 24, 48, and 72 h post one-visit endodontic treatment. The statistical analysis was done using Kruskal-Wallis, Mann-Whitney, and Freedman tests (P<0.05).

**Results:**

At all times, there was significant difference between the Ibuprofen and Zintoma (P<0.05) and also between the Ibuprofen and placebo (P<0.05). However, there was no significant difference between Zintoma and the placebo in any of time intervals (P>0.05). No side effects were observed.

**Conclusion:**

The obtained results of the trial revealed that prophylactic use of 2 gr Zintoma is not an effective pain relieving agent.

## Introduction

Pain control during and after endodontic treatment is one of the most important issues in endodontics [1, 2]. It has a high occurrence of 25-40% [3, 4]. Several factors influence this pain: the pulpal status, patients’ anxiety, existence of pretreatment pain, and manipulation of periapical tissues [3, 5-8]. Pain following endodontic therapy is often linked to the inflammatory process as well as additional central mechanisms [9, 10]. Many medications are prescribed in dentistry to control/reduce the pain. These drugs/materials include opioid and non-opioid analgesics, non-steroidal anti-inflammatory drugs (NSAIDs), benzo-diazepines and corticosteroids, and even MTA. [7, 11-16].

NSAIDs are generally considered to be the most effective treatment for inflammation and hence inflammatory pain. However, the many adverse effects, most important of which are gastrointestinal and cardiovascular, may outweigh the benefits [17]. That is why an increasing numbers of patients are searching for alternative forms of pain management, with minimal side effects. *Zingiber officinale (Z.officinale)*, commonly known as ginger is an anti-inflammatory agent for musculoskeletal diseases. Recently, there has been a lot of research performed on using the active ingredients of ginger and understanding it pharmacological effects in patients [18]. *Z.officinale* is a complex mixture of pharmacological compounds containing several hundred known constituents, including gingerols, beta-carotene, capsaicin, caffeic acid, curcumin, and salicylate [18-20]. It has been suggested that some of its chemical constituents, including gingerols, shogaols, paradols, and zingerone, have demonstrated anti-inflammatory actions, inhibiting leukotriene synthesis, as well as the activity of cyclooxygenase enzymes (COX-1 and COX-2), production of interleukins (Il-1 and Il-12), [21]. In addition, it has been suggested that *Z.officinale* and it’s constituents-particularly shogaols-have agonize vallinoid (capsaicin) receptors TRPV1, which are involved in the central and peripheral processing of noxious stimuli [18].

There are a lot of studies conducted on the prophylactic effects of the prescribed chemical drugs on post endodontic pain [12, 13, 22], in which there has not been dealt with herbals such as *Zintoma*. Direct and indirect capabilities of Z. Officinale for pain management are well documented [18, 21]; therefore, this randomized double-blind clinical trial study was designated to compare the prophylactic effect of *Zintoma* (a derivative of Ginger) and Ibuprofen on post endodontic pain related to one-step treatment of molar teeth with irreversible pulpitis. 

## Material and Methods

This study has been independently reviewed and approved by the ethics committee of Babol University of Medical Sciences (Ethical Code: 91-3013), and has been submitted on the website of Iranian Registry of Clinical Trial (ID No: IRCT138903174116N1). The initial sample size has been determined based on the power of 0.9 and the type I error of 0.05, with 30 subjects in each group (totally 90). The inclusion criteria included: healthy persons (ASA I or II), age 18 to 65, active pain in one of the mandibular molars, prolonged pain to cold test, spontaneous pain reported (at least 54 based on Heft-Parker Visual Analogue Scale (VAS), normal priapical view on radiographs, the patients ability in reading, comprehending and filling out the VAS form. The samples with the following criteria were excluded from the study: pain in more than one tooth, the existence of contributory medical history, allergy to lidocaine, NSAIDs or ginger, pregnancy or lactation, use of any sedatives or analgesics within the past 24 h, non-restorable tooth, tooth that was over instrumented or overfilled during treatment and where the patient was unable to comprehend the protocol of the study or sign the informed consent. The clinical diagnosis of irreversible pulpitis was done through the intense or prolonged reaction to cold stimuli(Roeko; Coltene Whaledent, Langenau, Germany) for at least 10 sec, and the existence of reaction to the electric pulp tester (Element Diagnostic Unit; SybronEndo, Glendora, CA).

After explaining the nature, purpose of the study and any probable risk and side effects of drug/treatment to the 90 participants, the written informed consent was obtained. Before taking the drugs, the patients were asked to record their pain intensity based on VAS as baseline, which was taught to the participants. Afterwards, the patients were randomly aligned into 3 groups of Ibuprofen, *Zintoma*, and placebo, using the “simple randomized allocation” method. It means each subject entering our study was allotted to one group by sequence.

The three drugs had been prepared by a pharmacologist in the same color and size capsules made of hard gelatin, and put in similar specially encoded pockets. The pharmacologist was in charge of encoding the capsules, in a way that neither the operator (Final year postgraduate student of Endodontics) nor the patient was informed of the drug. According to the group dedicated, each of the subjects was given a pack of the drugs along with Acetaminophen 325 mg (Aria Pharmaceutical Co, Tehran, Iran). The design of using drug for each group was as follows:

*Ibuprofen Group:* 1.5 h before the treatment, 2 placebo capsules and 0.5 h before the treatment, 2 capsules, one of which including placebo and the other having 400 mg Ibuprofen (Hakim Pharmaceutical Co, Tehran, Iran). Placebo capsules were given in this group to mirror the number of capsules given in the *Zintoma* group.

*Zintoma Group:* 1.5 and 0.5 h before the treatment, each time two 500-mg capsules of *Zintoma* (totally 2 g) given. Active ingredient in each 500-mg capsule is 2.5 mg, (Goldarou Pharmaceutical Co, Isfahan, Iran).

*Placebo Group:* 1.5 and 0.5 h before the treatment, each time 2 placebo capsules (Lactose powder) were given to the participants.

In case of the rescue drug (acetaminophen), the patients were asked to record the time and amount taken. All the patients were treated by one practitioner who was a final year postgraduate student of endodontics from September 2010 until April 2011. 

Half an hour after taking the second dosage of the drug, one cartridge of anesthetic solution including 2% lidocaine with epinephrine 1:200000 (Persocaine-E; Darupakhsh, Tehran, Iran) was injected for inferior alveolar nerve block (IANB). Root canal therapy started 15 min after anesthesia injection, if lip numbness had been obtained as the first sign of IANB success; otherwise the same injection was repeated. If the patient had recorded any pain or sensitivity to cold after IANB and before the start of endodontic therapy or any time during the treatment, complementary anesthesia techniques (buccal infiltration, intraligamentary and eventually intrapulpal injection respectively) were used. After preparing the access cavity, isolation with rubber dam and canal negotiation, working length determination was performed with apex locator (Root ZX, Morita Corporation, Tokyo, Japan) and was verified through PA radiography taken with bisected technique (1 mm shorter than radiographic apex). The primary canal cleaning and shaping was done by #10 and #15 K-Files (Dentsply Maillefer, Ballaigues, Switzerland) and #20, 25 and 30 K-Flex files (Kerr/Sybron, Romolus, MI) in pressurless crown-down technique. Canal irrigation was performed with 2.5 % NaOCl. The coronal areas of the canal were enlarged with #2 and #3 Gates Glidden and then #15 to #35 Mtwo rotary files, canals were prepared sufficiently. After completion of cleaning and shaping, the canals were dried with paper point (Aria Dent, Tehran, Iran) and filled with nonstandard gutta-percha (Meta Dental Corp., Chungju, Korea) and sealer AH26 (Dentsply DeTrey, Konstanz, Germany)with cold lateral condensation technique.

The access cavity was sealed with the temporary restoration (Coltosol; Asiachimi-Teb, Tehran, Iran) and patient was referred for the permanent filling of the tooth.

The VAS form was filled out by the patients immediately after temporization and then in 4, 8, 12, 24, 48, and 72 h, subsequently. Moreover, they were asked to fill out another form to record the time and amount of rescue (acetaminophen), if they had used it. The patients who had to take analgesic other than acetaminophen, because of intense pain or any other, were omitted from the study and replaced with other ones.

The data were analyzed through software SPSS 16 and Kruskal-Wallis, Mann-Whitney, and Freedman tests. The level of significance was 0.05. It also should be mentioned that there was no conflict of interest in this study.

## Results

Out of 90 patients entering this study, only 72 patients (80%) returned the questionnaires regarding post endodontic pain, meaning that the rest 18 subjects were lost to follow. The data related to number, age, gender, type of tooth, and initial pain score of each group has been shown in the Table 1.

**Table 1.** The number of the patients in each group, gender distribution, average age, type of tooth, and initial pain score

Group(n)	Age (year) [Mean (SD)]	Gender (n)		Molar (n)			Initial Pain Score Mean (SD)
		Male	Female	First	Second	Third	
**Ibuprofen (27)** ******	34.31 (10.35)	15	12	10	10	7	83.63 (20.48)
***Zintoma*** **(24)** ******	35.96 (9.89)	13	11	9	7	8	86.41 (22.78)
**Placebo (21** ***) ***	35.94 (9.99)	10	11	8	8	5	90.11 (21.72)
**Total (72) **							*P *= 0.61

There was no significant difference observed in terms of age, gender, the number of the first, second, and third molars in any of the groups and initial pain score (*P*>0.05). Furthermore, there was no significant correlation between gender and tooth type with perceived post endodontic pain. 

In the comparison between the post endodontic pain scores, at all times of the study, there was no significant difference between the three groups (*P*>0.05).

**Figure 1.**
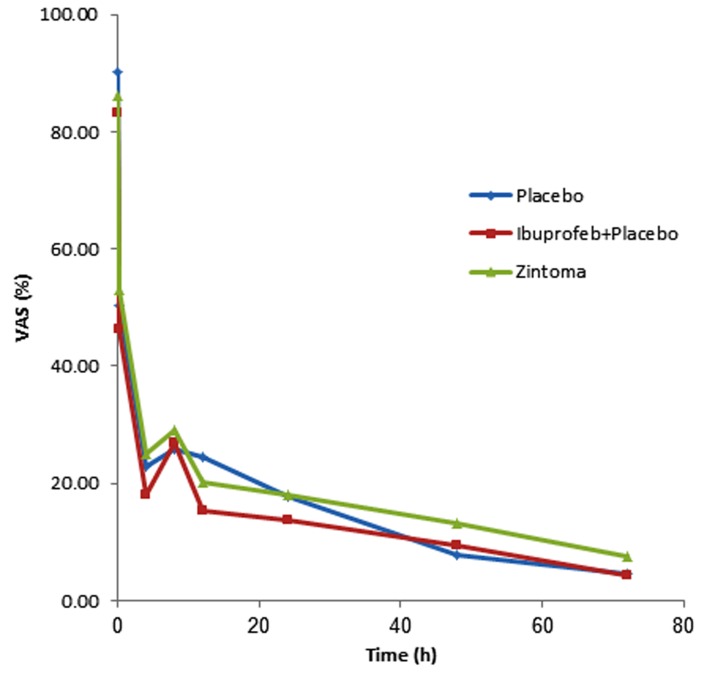
Pain reduction in three groups

At all times there was significant difference between three groups (*P*<0.05). In the two by two comparison between the groups at all times, the significant statistical difference was seen between the Ibuprofen and the *Zintoma *groups (*P*<0.05) and Ibuprofen and the Placebo groups (*P*<0.05). The pain score difference in each group was significant in different times (*P*<0.0001) (Figure 1).

The perceived pain by the patients in each of the three groups had been significantly reduced after the endodontic treatment (*P*<0.05). There was no significant statistical difference observed as a result of taking acetaminophen by the patients in the three groups (*P*>0.05). Statistical analysis related to the rescue drug (Acetaminophen) showed no significance (*P*>0.05).

## Discussion

Having assessed the tools for pain analysis, Coll *et al*. showed the appropriateness of the numerical VAS [23]. This scale is a 100 mm axis on which 0 indicates no pain and 100 shows the maximum pain possible. Numerical VAS is simply applied, analyzed and repeated. Moreover, it is not affected by the gender of the patient [24]. The second pain analysis scale is categorical VAS, which has five groups of pain including none to mild, mild to moderate, moderate to strong, strong to intense and intense to maximum possible. The first section itself is divided into faint and weak subgroups. Although simple, this scale is reliable and repetitive; but, each group of it contains great spectrum of pain intensity and quality [25]. Hence, the third scale, Heft-Parker VAS which is actually the combination of the first two ones, was introduced [23, 24]. In the present research, Heft-Parker VAS was applied to estimate the patient’s pain, before and after the endodontic treatment. Most of the previous studies had also used this scale to analyze the pain perception of the patients [3, 4, 12, 17, 26-28].

Besides responding to the cold test and electric sensitivity for clinical determination of the pulp status, bleeding or not, after the preparation of the access cavity, is considered as the golden standard in the pulp status diagnosis [29-34]. In this research, visual inspection and the existence of bleeding after the access cavity preparation was applied to confirm the pulp vitality, and consequently the patients whose tooth pulp showed no pulp bleeding were omitted from the study.

Some chemicals such as drugs from benzodiazepines, non-opioid analgesics, and opioids groups have been utilized prophylactically to decrease post endodontic pain, among which, NSAIDS, especially Ibuprofen has a noticeable role [26-28, 35, 36]. Having analyzed different dosages of Ibuprofen, Seymour *et al*. had concluded that a dosage of 600 mg has the highest effect as a painkiller in comparison with the dosages 200 and 400 mg [37].

Although in this research, like the Seymour’s trial there was just a single dosage of Ibuprofen (400 mg) analyzed, the acceptable effect of the drug, was also determined. In the study by Attar *et al*., a single dosage of 600 mg Ibuprofen had reduced the post endodontic pain more than the placebo, which is in line with the result of this research [35]. In a study by Gheshlaghi Azar and Kheradmand, the effect of celecoxib on pain reduction in endodontic treatment was even more than Ibuprofen [6]. But in our study, *Zintoma *was less effective than Ibuprofen. It should be mentioned that in the study by Gheshlaghi Azar, apart from the teeth with irreversible pulpitis, the necrotic teeth were also analyzed. Besides, in the study by Menke *et al*, 600 mg Ibuprofen had reduced post endodontic pain more than 400 mg Etodolac [38]. Of course, in the last two studies, the necrotic teeth were also included. The result of study done by Ehsani *et al*, to assess the role of prophylactic Ibuprofen and N-acetylcysteine (NAC) on the level of cytokines in periapical exudates and the post-treatment pain, showed that NAC can be a substitute for Ibuprofen in the management of post endodontic pain [39], whereas in our study, Ibuprofen was significantly more effective than the study drug. In another study for assessment of prophylactic effect of intraligamentary injection of 0.4 mL piroxicam (feldene) for the management of post-endodontic pain in molar teeth with irreversible pulpitis, the decrease in the intensity of post-treatment pain between the groups of piroxicam and placebo (same amount of lidocaine) was very significant [9]. In the study of Arslan *et al*, done to evaluate effectiveness of tenoxicam and Ibuprofen for pain prevention following endodontic therapy in comparison to placebo, prophylactic administration of a single dose of 20 mg tenoxicam or 200 mg Ibuprofen before root canal therapy provides an effective pain reduction at 6 h. Superiority of Ibuprofen to placebo was also confirmed in present research. Because of the advantages of tenoxicam, the authors suggested it may be useful as a prophylactic analgesic when post-endodontic pain is anticipated [12], whereas *Zintoma* did not show effectiveness for pain relief in our trial may be due to its short -term usage. In another study done by Rogers *et al*, to compare the effect of intracanal use of ketorolac tromethamine and dexamethasone with oral Ibuprofen on post endodontic pain, no significant differences were demonstrated between Ibuprofen and either dexamethasone or ketorolac tromethamine which prophylactically had been used intra canal. Although Ibuprofen pain ratings were less than the placebo at all-time points [22], that is in line with the results of the present study.

In the research done by Jalalzadeh et *al*, the prophylactic effect of prednisone was compared to placebo in terms of the endodontic pain, in which prednisone was more effective [4]. Mehrvarzfar *et al*. assessed the effect of supraperiosteal injection of dexamethasone on postoperative pain. According to their results, dexamethasone was considerably effective on controlling the severity of pain during the first 24 h; in contrast, there was no difference between dexamethasone and placebo groups 48 h after the first appointment. They concluded that a single dose of dexamethasone infiltrated around the apex of a tooth with irreversible pulpitis could be effective in reduction or prevention of postoperative endodontic pain during the first 24 h [40]. Up together, the noticeable differences observed in previously-mentioned trials are not unusual, probably because of different setting in case of drug dose, teeth involved, sample size etc.

In our electronic search, all of the previous well-documented researches found regarding the analgesic effect of *Z.Officinale* were related to either clinical chronic or experimentally induced acute pains, none of which had been conducted to assess the analgesic effect of *Z.Officinale* in dental practice, [41-46]. That is why we conducted the present study as the first academic trial in dentistry field to evaluate the analgesic effect of *Z.Officinale* on acute pain resulted from pulpitis.

*Zintoma* is an analgesic that is highly applied in medical science and has the least side effects. In most of the previous studies carried out on *Zintoma* such as the studies by Haghighi *et al*., Altman *et al*. and Bliddal *et al*., chronic use of the drug had significant difference with Ibuprofen in reducing the pain caused by osteoarthritis [41-43].

In this study, which aimed at analyzing the short-term effect of *Zintoma*, it could not present its anti-inflammatory and hence painkilling effect, and that can be why the findings indicated that the effect of *Zintoma* on post endodontic pain had no significant difference with the placebo. Another reason to mention for the little effect of *Zintoma* is related to the formation and continuation of all the inflammatory pains, such as the pain caused by tooth pulpitis, there have been a set of inflammatory mediators such as prostaglandins, serotonin, and histamine, with their synergic effect on each other [47, 48]. However, a drug such as *Zintoma*, that reduce the production of new mediators which are specially of prostaglandin type, not only doesn’t prevent the action of previously-existent mediators of any type, but also are less effective on the activity of other types of inflammatory mediators, and as a result, might have no significant effect on the reduction of inflammatory pain. Hence, the patients who have the inflammatory pain, before the embark of the treatment, may use less prophylactic effect of this anti-inflammatory drugs, in short period, in comparison with those who don’t have the pain beforehand. This could be one of the reasons for the difference observed between the results of the various studies. In the present study, the hand and rotary instruments were applied using crown-down technique. Since among of the effective factors on the post endodontic pain is the extruded debris behind the apex. It has been shown that this technique would lead to the lesser extrusion of the debris behind the apex [49].

It should be mentioned that use of placebo in this study had no conflict with ethical issues; since all the patients were informed of the procedure. Furthermore, it has been scientifically proved that the placebo along with the full pulpectomy of the teeth could cause pain reduction to 71% [35]. In the present research, the patients were monitored for 72 h and there was no side effects reported for *Zintoma*.

Due to a paucity of well-conducted trials, evidence of the efficacy of *Z.* *officinale* to treat pain remains insufficient. However, the available data provide tentative support for the anti-inflammatory role of *Z. officinale* constituents, which may reduce the subjective experience of pain in some inflammatory conditions. Further trials, particularly in dentistry field, therefore, seem to be warranted.

## Conclusion

Regarding the results of this double-blind randomized clinical trial study, it can be concluded that the drug regimen of *Zintoma* (1 gr, 1.5 h and 1 gr, 0.5 h prior to commence of endodontic treatment is not effective for post-treatment pain relief in molar teeth with irreversible pulpitis. It is recommended that further studies be done as for this drug, so that an appropriate dosage of *Zintoma* could be determined for endodontic treatments.
